# Impact of contraception and IVF hormones on metabolic, endocrine, and inflammatory status

**DOI:** 10.1007/s10815-020-01756-z

**Published:** 2020-03-25

**Authors:** Ayla Coussa, Hayder A. Hasan, Thomas M. Barber

**Affiliations:** 1 Division of Biomedical Sciences (T.M.B.), Warwick Medical School, Clinical Sciences Research Laboratories, University of Warwick, University Hospitals Coventry and Warwickshire, Clifford Bridge Road, Coventry, CV2 2DX UK; 2 Department of Clinical Nutrition & Dietetics, University of Sharjah, City University, Muwailih, PO Box 27272, Sharjah, United Arab Emirates

**Keywords:** Infertility, IVF, Pregnancy, Assisted reproduction, Gestational diabetes

## Abstract

Assisted reproductive technologies (ART) represent commonly utilized management strategies for infertility with multifactorial causes (including genetically predisposed diseases). Amongst ART, in vitro fertilization (IVF) is the most popular. IVF treatment may predispose the mother to increased risks and complications during pregnancy, and there may be adverse fetal outcomes. Hormonal therapies, including oral contraceptives, may impair glucose and lipid metabolism, and promote insulin resistance and inflammation. IVF treatment involves administration of reproductive hormones, similar in composition but in much higher doses than those used for oral contraception. The provision of IVF reproductive hormones to mice associates with glucose intolerance. In addition, the physiological and hormonal changes of pregnancy can trigger an inflammatory response, and metabolic and endocrine changes. There is controversy regarding the potential effects of IVF hormonal therapies in the promotion of diabetogenic and inflammatory states, additional to those that occur during pregnancy, and which may therefore predispose women with IVF-conceived pregnancies to adverse obstetric outcomes compared with women with spontaneously conceived pregnancies. This review summarizes the limited published evidence regarding the effect of IVF-based fertility therapies on glucose homeostasis, insulin resistance, cardio-metabolic profile, and markers of inflammation.

## Introduction

Infertility is a global health concern and affects 20% of couples of reproductive age. The use of assisted reproductive technologies (ART), including primarily in vitro fertilization (IVF), is becoming more prevalent. Oral contraceptive hormones commonly associate with gastrointestinal side effects from changes in gut microflora, in addition to possible adverse effects on glucose and lipid metabolism, which in turn may promote insulin resistance and inflammation. The concentration of reproductive hormones is much higher when used for IVF than oral contraception. However, confirmation of the safety of IVF (both maternal and fetal) remains tenuous. Previous studies have focused mainly on risk of obstetric complications and fetal outcomes in IVF-conceived pregnancies, with relatively few studies investigating the possible effects of IVF hormones on maternal metabolic, endocrine and inflammatory status, and the relationship to the known diabetogenic and atherogenic effects of pregnancy. Regarding the inflammatory response, the intestinal microbiota is of particular interest, given its central role in the modulation of immune-regulation (including during pregnancy), and insulin sensitivity. It would be desirable to have reliable predictive factors for possible metabolic, endocrine, and inflammatory sequelae of IVF therapies. Such factors could help prevent, or at least identify, at an early stage potential maternal and/or fetal complications, such as onset of gestational diabetes. In this brief review, we outline existing literature regarding the potential adverse maternal and fetal outcomes of exogenously administered reproductive hormones (oral contraception and IVF) on glucose and insulin homeostasis, metabolic profile, and inflammatory status.

### Infertility

Infertility is defined as the “inability to conceive after 12 months of unprotected intercourse, and 6 months for women 35 years of age and older” [1]. Globally, 15–20% of couples are infertile, and this corresponds to 35% female infertility, 30% male infertility, and 20% combination of the two, and the remaining 15% of cases corresponds to idiopathic or unexplained infertility [2, 3].

Many factors are associated with infertility in women. Mechanical impairment of the reproductive system accounts for about 35% of female infertility and includes damaged or blocked fallopian tubes, fibroids, and endometriosis. Age has a significant impact on female fertility, affecting both quality and quantity of eggs: reproductive age peaks in the 20s and early 30s and starts to decline after the age of 35 years [1]. Ovulation and hormonal-related disturbances are common causes of female infertility. Polycystic ovary syndrome (PCOS) is the most common endocrine disorder in women of reproductive age and is associated with obesity, hyperinsulinemia, and insulin resistance [4]. Although frequently co-existent with PCOS, there is evidence to support the notion that overweight and obesity are independent contributors to female infertility, mediated through adverse effects on reproductive hormones, manifesting as anovulation [5]. Thyroid dysfunction is also a common endocrine disorder to impair menstrual cyclicity and female fertility [6]. In men, fertility is adversely affected by advanced age, smoking, and obesity, similar to women, although age-related effects are less pronounced, with fertility only declining in men after the age of 50 years [7].

## Obstetric risks and complication of IVF therapies

### Risks of IVF treatment on maternal outcomes

Despite a steady increase in the medical treatment of infertility with ART, there is still a lack of published evidence on their safety. Compared with other ART treatments, IVF treatment predisposes to increased maternal risks and complications, since there is alteration of the normal physiological development of pregnancy during IVF. Furthermore, use of stimulating agents can also adversely affect pregnancy outcome, including association with ovarian cysts, ovarian enlargement, and ovarian hyper stimulation syndrome (OHSS) [3]. Ectopic pregnancy is twice as common, and pregnancy loss after 12 weeks is more prevalent with IVF-conceived pregnancies [8]. Furthermore, multiple pregnancies account for 25% of IVF-conceived pregnancies [9]. However, although twin and triplet pregnancies have a higher complication rate overall compared with singleton ones (including preeclampsia, gestational diabetes mellitus (GDM), thromboembolism, and preterm delivery), complication rates overall are similar between IVF-conceived and spontaneously conceived pregnancies [10, 11]. Future studies should focus on obstetric outcomes with IVF-conceived pregnancies in larger cohorts, well matched with spontaneous pregnancies, including those that result in multiple pregnancies.

One reason for higher rates of maternal adverse outcomes with IVF may relate to the frequent categorization of women undergoing IVF as “high risk,” as they usually present with advanced age, high BMI (> 30 kg/m^2^), or a pre-existing medical condition such as PCOS [12]. A higher prevalence of spontaneous abortion occurs in IVF-conceived pregnancies in women who are also obese and/or have a history of PCOS [13]. IVF-conceived pregnancy is a “high-risk” intervention with increased risk for maternal and obstetric complications. These include miscarriage, vaginal bleeding, frequent hospitalization, GDM, gestational hypertension, and preterm labor [14, 15]. However, there is some controversy in the literature regarding the actual risk of adverse obstetric and maternal outcomes with IVF: in a large retrospective study by Kozinszky et al. [10], data did not show increased rates of obstetric complications with IVF-conceived pregnancy. Cesarean section is more common with IVF. Women may consider IVF-conceived pregnancy as “precious” after many years of infertility and choose cesarean section to prevent perceived complications from a natural delivery, and not necessarily because of medical necessity [16]. Compared with spontaneously conceived pregnancy, women with IVF-pregnancies are more likely to develop GDM. This association remains following adjustment for maternal and gestational age, and parity [17]. It is possible that the increased risk for GDM in IVF-conceived pregnancies may stem from association with prenatal obesity or maternal PCOS (conditions that are not always specified) [18]. Furthermore, association of IVF with GDM may develop indirectly from the effects of IVF therapy on body fat accumulation, or directly from the procedure itself, through incompletely understood mechanisms. IVF may also associate with increased risk of breast and ovarian cancer post-IVF, although this association remains poorly described and more studies are needed [19].

### Risks of IVF treatment on fetal outcomes

Risk of congenital malformation was shown to associate with IVF, especially with multiple pregnancies. Mixed reports exist regarding fetal outcomes of IVF. While some studies suggest that IVF may predispose to intrauterine growth retardation, fetal anomalies, birth defect, and perinatal mortality [20, 21], others show no difference in fetal outcomes between spontaneous and IVF-conceived pregnancies [22, 23]. In one study, it was shown that IVF-conceived children are predisposed to obesity, insulin resistance, type 2 diabetes mellitus (T2D), and cardiovascular disease in adulthood [24]. Further prospective studies are required to clarify adverse effects of IVF on offspring, regarding both fetal development and longer term effects that manifest in adulthood.

## Does IVF therapy influence glucose homeostasis?

During early pregnancy, fasting plasma glucose level is similar to that of non-pregnant women and usually remains constant throughout pregnancy. However, although the level of fasting serum insulin is similar to that in non-pregnant women during the first trimester, fasting serum insulin levels increase significantly during the second and third trimesters [25]. Insulin action is also 50–70% lower (measured by hyperinsulinemic-euglycemic glucose clamp technique) during the second and third trimesters compared with the first trimester [26]. Insulin resistance drives increases in serum insulin during mid-pregnancy, reflected by increases in the homeostatic model assessment of insulin resistance (HOMA-IR), and decreases in the quantitative insulin-sensitivity check index (QUICKI) [25]. The diabetogenic effect of pregnancy stems from impairment of insulin sensitivity, and increased beta-cell activity in response to a greater requirement for insulin. This phenomenon occurs primarily during the second trimester, or late gestation [27]. Although insulin resistance plays an important role in the etiology of numerous adverse outcomes during pregnancy (such as GDM, preeclampsia, and miscarriage), the mechanisms implicated remain incompletely understood [25].

Under normal physiological conditions, estrogen and progesterone (gestational or maternal hormones) rise linearly during pregnancy and play crucial roles in supporting pregnancy and normal fetal development. Gestational hormones also play important roles in insulin homeostasis. While estrogen enhances insulin release and binding to its receptor, progesterone actually reduces insulin binding to its receptor and hence impairs glucose transport. Therefore, the diabetogenic effect of pregnancy relates to the rise in serum levels of these hormones along with the other placental-related (including lactogen, human chorionic gonadotrophin (HCG), human placental lactogen, growth hormone, and cortisol), which results in reduced insulin sensitivity, hyperinsulinemia, and impairment of “pre-implantation environmental state” [27, 28].

Studies on oral contraceptive therapies (estrogen and progesterone combination) report different findings in relation to their impact on glucose metabolism and insulin homeostasis. Impairment in insulin sensitivity and glucose tolerance was commonly experienced with the use of oral contraceptives and evidenced by higher glucose and insulin levels [29, 30]. While some research findings suggest that insulin resistance is induced by progesterone, others suggest that this is likely estrogen-related, and that progesterone only affects the half-life of insulin [31, 32]. Given that the dose of gestational hormones administered with IVF is higher than that used for combined oral hormonal contraception, it is not possible to extrapolate glycemic and metabolic effects of such therapies to those used for IVF. Interestingly, in mice models, IVF associates with glucose intolerance [33]. However, human data is severely limited in relation to the effects of IVF on insulin and glucose homeostasis during early pregnancy. Further studies are required to explore the potential for IVF-related hormonal therapies to augment the diabetogenic effect of pregnancy. Such data will likely provide further insight into early predictors of GDM.

## Does IVF therapy increase maternal cardiovascular and inflammatory risks?

The pregnancy-related inflammatory response is induced by physiological and hormonal changes, and detectable as early as embryo implantation [34]. Gestational hormones play an important role in the synthesis of inflammatory markers [35]. C-reactive protein (CRP), one of the commonly measured inflammatory markers, appears to increase with use of oral contraceptives, mainly in women below 35 years [36]. Based on this observation, it is hypothesized that IVF-related treatments may also stimulate an inflammatory response [34]. Increased serum levels of high-sensitivity CRP (hs-CRP), a more precise CRP assay of inflammation, has also been reported with increased age and BMI, factors that both associate with infertility, and often present in those seeking IVF treatments [37]. Furthermore, obesity associates with inflammation and is itself often an independent cardiovascular risk factor in obese women who use oral contraceptive therapies.

In addition to inflammatory response, pregnancy also induces changes in lipid and lipoprotein metabolism, usually evident following the first trimester. While some studies report an increase in all lipid parameters during pregnancy [38, 39], others show only a significant increase in triglycerides (TG) and very-low-density lipoprotein (VLDL), and a decrease in low-density lipoprotein (LDL) [40]. Impairment in the maternal lipid profile may predispose to pregnancy-induced hypertension, preeclampsia and GDM, and fetal macrosomia and cardiovascular diseases [39]. Hypertriglyceridemia results from pregnancy-related increased body fat and lipolytic activity, required to support pregnancy and in preparation for breastfeeding [41].

Studies on the effects of oral contraceptive therapies on lipid profile report conflicting data with regard to changes in LDL level, but consistent data regarding increased TG levels [29, 42]. Elevated estrogen level triggers hepatic synthesis of lipids, with increased serum levels of TG and total cholesterol (T-Chol) [38, 43]. Therefore, hypertriglyceridemia is thought to be estrogen dose-related [28]. The literature is deficient regarding reported data on the effect of IVF hormonal therapies on lipid profile, and whether the latter augments the atherogenic nature of pregnancy.

## Does IVF therapy influence the gut microbiome?

The relationship between intestinal microbiota and metabolic health is very topical and of much interest. Changes in the gut microbiota may influence the development of much twenty-first century chronic illness, including diabetes mellitus, cardiovascular disease, and dyslipidemia [44]. The gut microbiota influences chronic inflammatory effects through mediation of leakiness of the gut lining [45]. A useful serum marker for this process is lipopolysaccharide-binding protein (LBP), an acute-phase protein. LBP binds bacterial compounds, including lipopolysaccharides (LPS), an outer membrane component of gram-negative bacteria that normally reside within the gut and form the microbiota. While strongly correlated with obesity, LBS is also negatively associated with insulin sensitivity [46, 47]. The presence of LPS (also called endotoxins) in the gut is a normal physiological phenomenon, but can become problematic when it crosses over a leaky gut wall into the circulation. Gut wall permeability (with leakage of LPS into the bloodstream) is likely influenced by stress, including a high fat/energy-dense diet, or use of hormonal therapies. This can result in “metabolic endotoxemia” [48, 49]. High levels of serum LBP strongly correlate with LPS and associate with insulin resistance, obesity, and T2D [46].

In pregnancy, gut microbiota experiences a reduction in species count and flora diversity from early to late, which may predispose to gestational inflammation and metabolic impairments. Given that body fat increases and insulin sensitivity decreases throughout pregnancy, this in turn may impact the immune system, inducing gut microflora disturbances [50]. Gut flora dysbiosis is associated with pregnancy-related complications, such as insulin resistance, preeclampsia, miscarriage, intrauterine growth retardation, and preterm delivery.

There is likely a role for oral contraceptives in the development of inflammatory bowel disease (Crohn’s disease and ulcerative colitis) [51]. Furthermore, gastrointestinal side effects commonly occur with oral contraceptive therapies. Oral estrogen and progesterone treatment has been shown to affect gut permeability, LPS signaling and cytokines-mediated inflammatory diseases [52, 53]. We speculate that changes in microflora, LBP, and LPS levels may occur with IVF-related therapies, given the higher dose of reproductive hormones used compared with oral contraceptives, and the stress of the procedure. Mediation of the inflammatory effects of IVF therapies may occur through changes in the microbiota and serum levels of LBP and LPS. Such effects may extend throughout the IVF-conceived pregnancy. There are currently no reported studies on the effects of IVF treatments and IVF-related pregnancies on lipopolysaccharide markers (such as LBP and LPS) and gut microbiota. Assessment of gut permeability during pregnancy (through IVF and spontaneous conception) would form a novel focus for future research.

## Does IVF therapy alter thyroid function?

Thyroid dysfunction impairs menstrual cyclicity, female fertility, and pregnancy outcome, and is classified as the second most common endocrine disorder in women of reproductive age [6]. Impaired thyroid function predicts poor IVF fertilization outcome, emphasizing the importance of treating abnormal thyroid levels at the preconception stage [54].

Estrogen has a significant impact on thyroid-stimulating hormone (TSH) secretion and thyroid gland activity. During pregnancy, “estrogen dominance” interferes with thyroid metabolism by stimulating hepatic thyroxine-binding globulin secretion, thereby reducing levels of free thyroid hormones [55]. There is also a suppression of serum TSH level throughout pregnancy, with the lower normal level in the first trimester [56, 57]. Furthermore, there is an increase in serum beta human chorionic gonadotrophin hormone during pregnancy (β-HCG; pregnancy indicator hormone), the effect being particularly pronounced in twin pregnancies. β-HCG has stimulatory effects at the TSH receptor and may drive over-production of thyroid hormones during pregnancy, and also contribute to suppression of TSH production [58]. Recurrent pregnancy loss, preterm birth, and placenta abruption have been associated with high TSH level [6, 54].

Oral contraceptives and pregnancy alter thyroid function in similar ways, probably through estrogenic effects. However, although the mechanisms are similar, pronounced changes in thyroid hormones occur in pregnancy compared with the use of oral contraceptive therapies. The difference in magnitude of thyroid effects between pregnancy and oral contraceptive therapies likely relates to exogenous estrogen therapy having a dose-dependent effect on increasing serum thyroxine-binding globulin and total serum thyroxin levels in those with normal thyroid function [43, 59].

Regarding IVF therapies, in addition to exogenous estrogen, gonadotrophin-releasing hormone (GnRH) is also administered, the latter having been reported to affect levels of thyroid hormones (likely through indirect stimulation of gonadotrophin release and increased production of estrogen) [60]. There is a lack of data in the current literature on thyroid status in IVF-conceived versus spontaneously conceived pregnancies. Given the potential for cumulative effects of estrogen-related thyroid dysfunction during IVF-conceived pregnancies, this should be a focus for future research.

## Conclusion

The increased risk of complications and adverse outcomes of IVF-conceived pregnancies remain contentious. However, maternal preconception characteristics are likely to play a role. This promotes the importance of screening high-risk populations at preconception, and early management of controllable factors to prevent possible obstetric complications and predict IVF success. The impact of pregnancy on metabolic, endocrine, and inflammatory parameters is well established. However, similar data in IVF-conceived pregnancies are lacking. Identifying early maternal metabolic and inflammatory biomarkers during IVF therapy and IVF-conceived early pregnancy may act as predictors for future maternal and fetal problems during the ensuing pregnancy. Such predictive factors could improve both pregnancy and fetal outcomes of IVF therapies.

## Abbreviation

ART: Assisted reproductive technologies
IVF: In vitro fertilization
GDM: Gestational diabetes mellitus
LBP: Lipopolysaccharide-binding protein
